# Current and Alternative Therapies for Nasal Mucosa Injury: A Review

**DOI:** 10.3390/ijms21020480

**Published:** 2020-01-12

**Authors:** Jegadevswari Selvarajah, Aminuddin Bin Saim, Ruszymah Bt Hj Idrus, Yogeswaran Lokanathan

**Affiliations:** 1 Tissue Engineering Centre, Faculty of Medicine, Universiti Kebangsaan Malaysia, Cheras, Kuala Lumpur 56000, Malaysia; jegadevswariselvarajah@gmail.com; 2 Ear, Nose & Throat Consultant Clinic, Ampang Puteri Specialist Hospital, Ampang, Selangor 68000, Malaysia; 3 Department of Physiology, Faculty of Medicine, Universiti Kebangsaan Malaysia, Cheras, Kuala Lumpur 56000, Malaysia; ruszyidrus@gmail.com

**Keywords:** nasal mucosa, wound healing, regenerative medicine, nasal injury

## Abstract

Nasal mucosa injury can be caused by trauma, radiotherapy, chronic infection such as sinusitis, and post sinus surgery. The rate of healing and its treatment are important in the recovery of patients especially in post sinus surgery, which introduces new injuries. In this review, the current knowledge in terms of the mechanism underlying nasal wound healing was initially discussed. The currently available treatment options for enhancement of wound healing following sinus surgery were discussed and these had included intravenous antibiotics or steroids, various nasal sprays, and nasal packing. In addition, emerging alternative therapies in nasal mucosa wound healing such as herbal medicine and the advancement of regenerative medicine therapies such as stem cells and their byproducts were also discussed. Despite the various available treatment options for wound healing in nasal mucosa, rigorous strong evidence of their efficacy is gravely warranted in order to recommend them as part of the treatment modality.

## 1. Introduction

Nasal diseases, particularly as a result of chronic inflammation and infection such as rhinosinusitis, significantly affect the quality of life of the patient [1]. Rhinosinusitis is a common nasal disease that affects approximately 5–15% of the general population [2]. Treatments such as antibiotics, nasal douche, steroid, and nasal sprays are commonly being prescribed to patients to eliminate infection, reduce inflammation, and revert the diseased mucosa to normal, functional respiratory epithelium. Another common treatment is a surgical intervention known as endoscopic sinus surgery (ESS), which is prescribed after the failure of conservative treatment following nasal diseases such as chronic rhinosinusitis and nasal polyposis [3].

However, mucosal damage is inevitable in this intervention and these damages may lead to serious known complications such as synechia, osteitis, or fibrosis, especially for difficult areas such as the frontal recess [4]. This can be due to several factors such as metaplasia of the mucosal lining due to chronic inflammation or secondary injury to the remaining healthy tissues during the procedure that can jeopardize the regeneration of the nasal mucosa [5].

The significance of ESS in otorhinolaryngology practice lead to an interest in understanding the post-surgical wound healing of the nasal mucosa. Unfortunately, to date, the majority of our knowledge is available in terms of wound healing, which concerns the repair of wounded skin or cornea [6]. In terms of the nasal mucosa, little is known about the physiological basis of the wound healing of this tissue [7]. Watelet et al. had compiled and discussed scientific literatures regarding nasal mucosa wound healing since a decade ago [8]. In this review, recent updates on the knowledge that had been accumulated were presented in terms of nasal mucosa physiology, and wound healing following nasal surgery management.

## 2. Nasal Mucosa Wound Healing

The nasal epithelium is made of a continuous layer of pseudostratified columnar epithelial cells that are separated from the lamina propria by a continuous basement membrane. Within the epithelium, four distinctive cell types can be found, which are the basal, goblet, ciliated, and non-ciliated columnar cells. In intact mucosa, nasal epithelial cell functions include mucus production and transport, resorption of surface fluid, homeostasis, and immune responses in concert with the underlying lymphatic and vascular tissue [7]. Damage to the nasal mucosa can be a consequence of trauma post-radiotherapy or post-surgery, and infection in chronic rhinosinusitis.

Following ESS, normal wound healing in nasal mucosa consists of four clinical different stages of the healing process, i.e., the stage of immediate cleaning of the operative cavity, mucosal transition, complete epithelialization, and tissue remodeling [9]. Physiologically, these four phases are correlated to the hemostasis, inflammation, proliferation, and tissue remodeling phases of wound healing that have been observed in in vivo models of nasal injury using rabbits [10] and rats [11].

Injury to the nasal mucosa causes hemorrhage. Hence, the first agenda of wound healing is to seal the leakage through the formation of a fibrin plug via the activation of the coagulation cascade [6]. Immediately following surgery, blood crusting can be observed dominating the nasal cavity in the first two weeks. At the end of the two weeks post-surgery, the operative cavity becomes clean in preparation for the next stage [12]. Similar crusting formation can also be observed through the gross morphology of the nasal wound in the rabbit [10] and rat [11] model.

Following the hemostasis phase is the inflammatory phase. The key aim of this phase is to clear infection at the wound site [6]. In humans, the operative cavities experience a mucosal transition in response to the mucosa removal within 3–10 weeks post-surgery. The transitions that can be observed include the mucosal edema, vesicles, granulation tissue, mini-polyps, fibrous hyperplasia, and adhesion. These will eventually be cleared to give way for the mucosal epithelialization process [12]. In vivo, sub-epithelium edema and infiltration of leukocytes were observed in a two-day post-wounding in rats [11] and a three-week post-wounding in rabbits [10].

Upon the clearance of pathogenic materials and dead immune cells, the proliferation phase follows [6]. The clearance of mucosal transition is followed by the mucosal epithelialization, which occurs within 11–14 weeks post-operation in humans [12]. Increased subepithelial fibrosis and epithelial thickness were noted on day 14 post-wounding in rats [11] while epithelial thickness in rabbits reached its peak in the four-week post-wounding [10].

The last phase of wound healing is tissue remodeling. In the nasal mucosa, this phase involves the differentiation of nasal epithelial into the specialized cells, goblet cells, and ciliated cells. In humans, changes in the subepithelial mucosa could be noted up to six months following the surgery [12]. In a rat model, epithelial differentiation into goblet cells and ciliated cells began on day 14 and completely restored to near-normal on day 28 [11]. In a rabbit model, the number of ciliated cells reached its peak in the five-week post-wounding [10].

## 3. Postoperative Complications of Endoscopic Sinus Surgery

ESS is a technique that utilizes endoscopic vision to enable the surgeon to reach the paranasal sinuses with minimal damage to the surrounding tissue [3]. It is one of the most common procedures performed in otorhinolaryngology and its success rate depends on the postoperative wound healing outcome [3].

Following the introduction of surgical injury, bleeding is anticipated due to the fact that the nasal cavity is rich with blood supply derived from the external and internal carotid arteries in this region [13]. Postoperative bleeding most commonly occurs within the first 24 h of the procedure but can be delayed for days or even weeks. In the event of hematoma formation within the nasal mucosa, its removal is necessary to prevent ischemia and fibrosis leading to the development of scarring [4].

Another common complication following ESS is tissue adhesion, which is also known as nasal synechiae. Nasal synechiae form when two moist, opposite surfaces inside the nose heal together, forming fused fibrous tissue that may block the normal airflow through the nose [14]. Due to its role in extracellular matrix (ECM) deposition and remodeling, the nasal fibroblast is thought to be responsible for nasal synechiae.

There is also a subgroup of patients with recurring chronic rhinosinusitis who have presented bone thickening known as osteitis. Osteitis is the thickening of the bone due to inflammation. Diseased mucosal can affect the viability of the bone underneath it. Over time, this poor viability can develop into bony inflammation that finally leads to the bone thickening. Little is known about post-surgical osteitis and the strategy to treat this condition [15].

Hence, major objectives of postoperative management of nasal wound healing aim to control postoperative bleeding, preventing adhesions, and expedite the healing process [4].

## 4. Post-Surgical Management of Nasal Wound Healing

A multitude of topical interventions and dressings has been used to facilitate the nasal mucosa wound healing following invasive sinus surgery. Interventions that have been used include intravenous antibiotics or steroids, nasal douching, and nasal packing to prevent infection and attenuate prolonged inflammation, thus collectively improving the nasal mucosa healing process.

### 4.1. Systemic Drug

Post-surgical management with systemic drugs involves the oral or intravenous delivery of steroids and antibiotics that are used to reduce the inflammation and infection that halts the progression of the wound healing following sinus surgery [16].

Among corticosteroids that were being used post-surgically were oral betamethasone [17] and oral prednisolone [18]. The use of systemic steroids in FESS perioperatively is arguable; however, recent systematic reviews and meta-analysis have revealed that the administration of systemic steroids, especially postoperatively, might be associated with the improvement of endoscopic scores and reduce the risk of recurrence among patients with chronic rhinosinusitis with nasal polyposis [16]. Jorrisen and Bachert investigated oral betamethasone (2 mg for seven days) followed by topical momethasone furoate sprays (200 µg b.i.d for six months) to be associated with reduced risk of sinusitis (RR 0.76, 95% CI 0.31, 1.90), and improved postoperative endoscopic score. On the other hand, postoperative administration of prednisone (oral, 30 mg for nine days) was observed to result in healthier sinus cavities [18].

Despite having most of the patients benefiting from systemic steroids in terms of wound recurrence prevention, a significant proportion of patients still experienced wound recurrence even in the immediate postoperative period [16]. Furthermore, long-term use of systemic steroids can lead to a plethora of adverse side effects [19].

There was little evidence to support the use of antibiotics in post-FESS patients [20]. A three-week course of amoxicillin and clavulanate potassium (375 mg, t.d.s. for three weeks) was shown to have no significant difference in terms of symptoms and endoscopic score as compared to the control [21]. A similar result was also demonstrated in a randomized, double-blind controlled trial with oral amoxicillin (250 mg, t.d.s for four weeks) [22]. Meanwhile, a short follow-up study reported that the two-week course of amoxicillin and clavulanate (625 mg, b.d) only improved nasal obstruction and drainage on the fifth day in regards to other symptoms and improved endoscopic scores at day five and 12 [23]. Due to the emergence of bacterial resistance against macrolide, the risks outweigh the benefits, and the reduced usage of antibiotics should be prompted.

### 4.2. Nasal Spray

Nasal spray is a method of delivering drugs, mainly steroids, into the intranasal space using an aerosol spray bottle. The main objective of post-surgical management with nasal spray is to modulate the inflammation phase of the nasal wound healing [24,25]. It is considered as the standard medical treatment for the control of the recurring sinusitis following surgical intervention [17]. Beclomethasone dipropionate, the first aerosolized topical corticosteroid, has been used clinically since the 70s. Numerous other aerosolized steroid preparations have been described in the literature including prednisolone acetate, mometasone furoate, triamcinolone acetonide, and fluticasone propionate [24]. Meanwhile, different types of intranasal steroid spray were reported to have different efficacy following post-FESS [26]. This has been demonstrated by the recent clinical trial (ClinicalTrials.gov: NCT02194062) that determine two types of intranasal steroid spray (fluticasone and budesonide) to reduce the incidence of polyposis in post-FESS chronic rhinosinusitis patients. Budesonide was observed to be superior (improvement in Sino-Nasal Outcome Test (SNOT-22) and Lund–Kennedy scores) than fluticasone [26].

Topical antibiotic and antifungal delivery using nasal spray were once considered to be the cornerstone of post-surgery management following ESS. This is due to the old paradigm that postulated that the inflammation seen in post-surgical nasal is a result of a microorganism infection in the sinus. In the age of the antibiotic-resistant microorganism, both therapies have generally fallen out of favor in terms of post-surgery management following ESS [24].

As the technology has developed, nasal spray is used to deliver bioactive compounds such as ECM component or coagulation cascade component. In the in vitro skin wound healing model, the ECM protein hyaluronan has been shown to enhance re-epithelialization [27]. Nebulized sodium hyaluronate administration into the nasal cavity has been shown to induce a faster recovery following ESS while maintaining the patient’s comfort throughout the process [28].

The idea of delivering coagulation cascade component into the nasal cavity centers around resolving the hemostasis phase to allow wound healing to progress [29]. A prospective study comparing the administration of aerosolized fibrin and non-absorbable nasal packing has revealed that crusting, adhesion, bleeding, granulation tissue formation, infection, and frontal sinus ostium stenosis after endoscopic surgery, as well as overall comfort, improve after using fibrin spray [30].

### 4.3. Nasal Packing

Simultaneous to the development of nasal spray is the use of biomaterials as a nasal packing. A major goal of nasal packing is to enhance postoperative wound healing by expediting the healing process, preventing adhesions, and control postoperative bleeding. Massey and Singh have published a thorough review of the biomaterials used in nasal packing [31]. Nasal packing is designed as a foam or sponge that can act as a tampon that is inserted into the nasal cavity to provide pressure to stop bleeding. Nasal packing can be absorbable or non-absorbable.

The non-absorbable packing has been used in sinus surgery for decades before the emergence of its absorbable variant [31]. Nasal packing can be a source of pain and discomfort for patients due to the removal process of the nasal packing. In many cases, its removal has been described by some patients as the most painful part of the entire procedure [32]. Thus, the development of absorbable nasal packing follows in the following decades.

In terms of absorbable nasal packing, several materials have been known to be used to fabricate them. In general, they can be divided into three categories: ECM protein, coagulation agent, and biopolymer. Such materials include gelatin, hyaluronan, fibrin, chitosan, cellulose, potato starch, carboxymethyl cellulose, polyurethane, and polyethylene glycol [31]. They have been fabricated into various forms including foams, gels, meshes, films, and powders. Although bioabsorbable nasal packing provides a better outcome in terms of preventing synechiae and halting epistaxis, its effect on wound healing enhancement is moderate to insignificant.

The earliest form of absorbable nasal packing is in the form of gelatin film [33] or foam [34]. When compared to the untreated nose, no significant differences were seen in terms of adhesions, granulation tissue, or edema outcomes [33,34].

Hyaluronan is one of the major components of the extracellular matrix that is known to enhance re-epithelialization in vitro [27]. Hyaluronan in gel form, MeroGel, has been extensively studied with a total of four RCTs conducted to our knowledge. When compared with the non-absorbable nasal pack, MeroGel performed better with respect to preventing adhesions at 4 and 12 weeks postoperatively in one study [35]. However, the other three earlier studies were not able to observe significant differences in terms of wound healing outcome such as postoperative edema and scarring between the treatment (MeroGel) and the control group [36,37,38]. Moreover, trials with crosslinked hyaluronan water-insoluble gel [39] and hydrogel [40] were reported to have performed better. Taken together, hyaluronan products seem to confer modest benefit with respect to the wound healing of nasal mucosa.

In terms of other biomaterials, two materials, fibrin [41] and chitosan [42,43], demonstrated superior wound healing and hemostatic properties in comparison to the non-absorbable nasal packing while cellulose [44], potato starch [45], carboxymethyl cellulose [46], polyurethane [47], and polyethylene glycol [48] remain similar to the untreated control or non-absorbable packing [31]. Table 1 summarizes the effect of absorbable nasal pack made with different biomaterials. In general, utilization of nasal pack is equivalent to the control.

As the field progresses, the postoperative management of nasal wound healing has shifted into the paradigm of a functional nasal pack. In this paradigm, biomaterials that were fabricated into an absorbable nasal packing were used as a delivery vehicle for known medications for wound healing such as steroids and antibiotics.

Silsos gel^®^ is a registered nasal packing that is made from silver sucrose octasulfate in association with potassium sucrose octasulfate, sodium hyaluronate, propylene glycol, carbomer, and water [49]. Silver is a well-known antimicrobial agent that is commonly used for cutaneous wounds. In a randomized, placebo-controlled trial, the patients who were treated with Silsos gel^®^ were reported to have performed better at the Sino-Nasal Outcome Test 22 (SNOT22) scale compared to the placebo group. Better mucosal integration has also been observed in the Silsos gel^®^ group endoscopically. The placebo contains the gel (carbopol and propylene glycol) without the silver. The study has successfully shown the efficacy of silver in nasal mucosa wound healing [49].

SinuBandFP is a 2 cm × 2 cm bi-layered thin film that is made up of fibrinogen and is fortified with a total of 160 µg fluticasone propionate. After application of SinuBandFP in the nasal cavity, the corticosteroid fluticasone propionate will be released over time. In a randomized controlled trial to investigate its safety and efficacy, the SinuBandFP group had demonstrated local safety, ocular safety, and no significant changes in urine cortisol after 24 h when compared to the SinuBand without the corticosteroid. In terms of efficacy, the SinuBandFP group did better in terms of polyp score, adhesion occurrence, and general pain [50].

Nasopore^®^ is a bioabsorbable nasal packing that is made of a fragmentable poly (DL-lactide-co-E-caprolactone) urethane. In a randomized, placebo-controlled study, Grzeskowiak and colleagues compared the efficacy of the Nasopore^®^ packing impregnated with either steroid (betamethasone) or antibiotics (ciprofloxacin) [51]; Nasopore^®^ impregnated with saline was used as a placebo. The study indicated a significant improvement with both steroid-eluting and antibiotic-eluting bioabsorbable packing on the postoperative healing process and patient satisfaction as compared to saline-soaked packing. Table 2 summarizes the effect of a functional nasal pack in the different outcomes of nasal wound healing. Nasal pack offers a drug delivery system that can be tailored according to the needs of the patient.

## 5. Complementary and Alternative Management of Nasal Injury

Management of nasal injury has been described in many cultural and religious records in the past. Due to the intimate relationship between the nasal mucosa and the external environment through the air that is breathed in, herbal medicine plays a large role in the management of the many nasal injury occurrences within different cultures [52].

### 5.1. Nasal Irrigation

The ancient Hindu practice of Ayurveda provides the earliest record of nasal irrigation [53]. The Ayurvedic scriptures list out a number of personal hygiene practices termed soucha. Among the Soucha, there is *jala neti*, also known as the practice of nasal irrigation [54]. According to the scripture, a higher state of meditation can be achieved by purifying the nose as clear breathing can lead to clear thinking. The simplest method of nasal cleansing was to sniff water from cupped hands and blow it out, which is also a step in the Muslim ablutions practice [53]. In modern science, data from RCT has demonstrated the importance of nasal irrigation in enhancing the wound healing of the nasal mucosa [55].

The precise mechanisms are still unknown but most experts think that it is due to the direct cleansing of the nasal mucosa, independent of the solution composition used [54]. This causes the mucus lining to be soft and dislodge. Furthermore, antigen and inflammatory mediators such as leukotrienes and prostaglandins that cause allergic reactions and can be removed by nasal irrigation. The composition of salt solutions can affect the effectiveness of nasal irrigation where the use of a lower concentration of salt and isotonic solutions will immediately reduce the microbial antigens significantly. On the other hand, it is shown that hypertonic solutions that are used can minimally influence the concentration of the microbial antigens.

Nasal irrigation with the addition of ions such as sodium and chloride can promote the integrity and function of epithelial cells. Moreover, the addition of magnesium will reduce eicosanoid metabolism by directly inhibiting the 5-lipoxygenase enzyme, encouraging cell repair, and limiting inflammation [55]. Magnesium also inhibits exocytosis of permeabilized eosinophils and reduces respiratory cells apoptosis in association with zinc [55].

### 5.2. Chinese Medicine

In Chinese medicine, herbal formulations are created to balance the “Yin-Yang”, which is based on traditional Chinese medicine (TCM) theory. The occurrence of diseases is thought to be the result of imbalance within the theory. In Asian countries such as China, nasal steroids and oral antibiotics are used along with herbs as an adjuvant treatment for post-ESS care. A study was done to investigate the safety and effectiveness of Zhu-Yuan decoction (ZYD) in the postoperative care of patients for FESS. In TCM theory, ZYD is used to treat Chinese medicine symptoms (phlegm and heat obstructing the sinus). The study has shown that ZYD administration has produced significant results that have similar safety and efficacy as intranasal cortisone. However, the study was short-term (lasting 12 weeks) and required the study of long-term effects and further study to elucidate the underlying mechanisms of ZYD [56].

### 5.3. Bee Propolis

Propolis is the material used by bees to build their hives. Synthesized by bees from plant resin, it has been demonstrated to have anti-inflammatory activity [57]. A study with rat models and nasal injury have revealed the reduction of inflammation and enhancement of healing of wounds of the nasal mucosa [58]. It has also been shown in a study that propolis reduces the severity of the inflammation and preserve both goblet cells and ciliary in nasal mucosa [58]. The exact mechanisms of nasal mucosa wound healing by propolis requires further study but its healing properties have been suggested to be due to its immune-stimulating effect where cytokine secretion capacity increases significantly during the treatment period in a time-dependent manner. Furthermore, propolis can stimulate a significant increase in ECM components during the initial phase of wound repair. Another study that looks into caffeic acid phenyl ester, a bioactive compound of propolis, has also revealed enhancement of wound healing in the nasal mucosa [59].

### 5.4. Curcumin

Among the spices, the medicinal properties of turmeric have been reported substantially [60]. In cutaneous wounds, curcumin has demonstrated anti-inflammatory and wound enhancement properties [61]. Utilizing the nasal injury rat model, curcumin has also been reported to reduce inflammation and enhance wound healing in the nasal mucosa [62]. This is due to a reduction of the inflammatory response in the nasal mucosa by inhibiting the cytokines production for the activation of macrophages and monocytes [62]. On the other hand, curcumin enhances the granulation tissue organization, which contains a higher number of smaller capillaries and myofibroblasts in a diabetic rat model [62].

## 6. Tissue Engineering and Regenerative Medicine

Tissue engineering is a branch of regenerative medicine that employs a multidisciplinary approach to achieve tissue repair and regeneration through the combination of three elements: stem cells, biomimetic scaffolds, and bioactive molecules. In the context of nasal mucosa regeneration, tissue engineering is promising in reaching the goals of regeneration.

### 6.1. Cell and Tissue Therapy

Stem cells have been the subject of interest in regenerative medicine since the dawn of the 20th century. In terms of wound healing, there were reports on its efficacy with skin [63] and corneal epithelium [64] wound healing. Utilizing the nasal injury rabbit model, Kavuzlu et al. implanted adipose-derived mesenchymal stem cell sheet onto the nasal mucosa to enhance its healing [65]. The implant resulted in better morphology, abundance, and density of the ciliated nasal epithelial cells. The mechanisms regarding the healing process have been suggested to be due to increased re-epithelization and stimulation of wound angiogenesis through the secretion of growth factors, cytokines, and collagen tissue as well as antioxidant effect through neutralization of reactive oxygen species [65].

Another study has attempted to utilize the aerosol delivery technique to deliver regenerative cells onto the injured tissue such as the works of Kardia and colleagues [66]. They successfully demonstrated an improvement of regeneration and repair in the respiratory tract of a rabbit upon delivery of aerosolized allogenic airway epithelial cells [66]. The regeneration and repair process involved rapid re-epithelialization of the denuded region where cell dedifferentiation, migration, proliferation, and re-differentiation occur for the repopulation of the tracheal epithelium. Furthermore, it has been suggested that the repair process is mediated by secretions of compounds such as growth factors, cytokines, and chemokines to induce tracheal epithelium repair [67].

In many surgical interventions, autologous tissue graft is considered as the gold standard. This can be observed in burn wounds [68], ligament injury [69], and osteoarthritis [70]. The use of autologous nasal mucosa grafts on rabbit has shown to improve re-epithelization. Utilizing light and scanning electron microscopy, Topdag et al. had demonstrated that the ciliary epithelium covered greater area, had more mature and sophisticated cilia, and had less hypertrophied epithelium in grafted tissue compared to the non-grafted tissue [6].

### 6.2. Tissue Scaffolds

Tissue scaffolds serve as a template that provides support to cells in engineered tissue. Scaffolds are fabricated in a way that resembles the native tissue, either through structural networks and geometries or biochemical compositions.

The biomaterial that is used to construct a scaffold can be of natural or synthetic origin. One of the types of natural tissue scaffolds is the decellularized matrix of native tissue. Decellularized tracheal extracellular matrix has been shown to positively influence the migration, differentiation, and function of respiratory epithelium in the mouse model of orthotopic tracheal transplantation [71].

Collagen and hyaluronan are two commonly used natural biomaterials for the ciliary differentiation of human respiratory epithelial cells [72]. In combination, bi-layered collagen-hyaluronate scaffolds have been shown to facilitate lung epithelial cell differentiation and mucin expression [73].

Tissue-engineered scaffolds can also be of synthetic origin. Fabrication of bi-layered trachea using autologous nasal respiratory epithelial cells and fibroblast seeded on titanium scaffold has successfully induced re-epithelialization within the sheep trachea [74]. On top of that, the work of a hybrid combination of natural and synthetic biomaterial for respiratory tissue engineering has shown promising results [75]. Rabiatul et al. utilized the technique of surface functionalization to electrospin polymethyl-methacrylate (PMMA), of synthetic origin, with collagen type 1, a natural polymer. The result had shown that the hybrid scaffolds were able to support respiratory epithelial cells attachment and promoted proliferation [75].

### 6.3. Cell Secretory Proteins, Growth Factors and Conditioned Medium

As an alternative to the stem cell differentiation hypothesis, the effect of stem cells on wound healing has also been hypothesized to be through the secretion of cytokines and growth hormones that activates resident cells in the tissue to regenerate. In the laboratory, cell-secreted factors can be found in the medium where the stem cells are cultured. Hence, upon collection, this medium is termed as conditioned medium.

As a result, the healing properties of the conditioned medium of stem cells have also been the subject of interest in regenerative medicine. Delivery of airway epithelium into the respiratory tract has been proven to improve its regeneration [76]. The secreted factors that are derived from the airway epithelium have also been demonstrated to accelerate the early repair of the tracheal epithelium [77]. The early repair might have involved reduced inflammation that is mediated by secretion of factors by the airway epithelium such as IL-10, which reduces the levels of pro-inflammatory cytokines (IL-1β and IL-6) in addition to secretion of other factors such as vascular endothelial growth factor α and mucin proteins [77].

Fibroblasts are the cells of the connective tissue that play supporting roles in the tissue where they reside, one of such is the synthesis of the extracellular matrix [76]. Nasal fibroblast conditioned medium has also been proven to enhance respiratory epithelial cells proliferation and migration by utilizing redundant tissues from turbinectomy procedures [77].

Mesenchymal stem cells and their secretory factors are also a subject of interest in the context of tissue regeneration. Conditioned medium from umbilical cord-derived mesenchymal stem cells has been shown to improve nasal epithelium healing both in vivo and in vitro [78]. In a study using adipose-derived mesenchymal stem cell secretions, the improved regeneration of airway tissue has been attributed to the stimulation of the epithelial to mesenchymal transition [79].

To support the notion for clinical application of human bone marrow-derived mesenchymal stem cell (MSC) secretome in regeneration for the treatment of idiopathic pulmonary fibrosis and other fibrotic lung disorders, Akram et al. tested the MSC conditioned medium (MSC-CM) with the human type II alveolar epithelial cell line A549 cells (AEC) and primary human small airway epithelial cells (SAEC) using an in vitro scratch wound repair model. They found that the MSC-CM in their study contained fibronectin, lumican, periostin, and IGFBP-7. They also demonstrated that the MSC-CM facilitated AEC and SAEC wound repair through stimulation of cell migration [80].

Recombinant protein technology has enabled scientists to synthesize individual growth factors. As a result, the study on the effect of individual growth factors in many of the human diseases has been made possible. The hepatocyte growth factor (HGF), which has been initially discovered as a mitogen for hepatocyte, is mainly produced by the mesenchymal cells [81]. It is also known to stimulate epithelial proliferation motility, morphogenesis, and angiogenesis in various organs through tyrosine phosphorylation of its receptor, c-Met. In the nasal mucosa, HGF has been shown to accelerate wound healing through the acceleration of re-epithelialization, and the augmentation of ciliogenesis [82].

Nerve growth factor (NGF) has also been reported to enhance epithelial healing following surgery [83]. An air-liquid interface utilizing nasal epithelium was used to test the effect of NGF on wound closure rates and expression of cell adhesion, tight junction formation, cell proliferation, and ciliogenesis-related protein. The study successfully demonstrated the acceleration of epithelial wound closure with NGF [84].

Insulin-like growth factor (IGF-I) is a hormone that has a similar molecular structure as insulin. Mainly secreted by the liver, IGF-I is important for both the regulation of normal physiology and several pathological states [85]. In vitro, IGF-I had demonstrated accelerated wound healing in human epithelial cell lines that were derived from the nasal, bronchial, and tracheal regions [86]. When IGF-I was incorporated into the hyaluronan-based nasal pack, it had demonstrated a selective improvement of healing rate in healthy sheep nasal mucosa tissue but not the sheep with inflamed tissue [87].

## 7. Plasma Therapy

Researchers have expanded their interest in studying plasma effect in medicine as some of the literature have suggested its beneficial effects including anti-inflammatory, anti-cancer, anti-microbial, and even tissue regeneration. Plasma is considered as the fourth state of matter, which is made up of partially ionized gas that contains electrons, radicals, energetic photons, and ions.

More importantly, the application of plasma therapy has been documented recently by Won et al. (2018) [88] by studying non-thermal plasma-treated solution (NTS) and its therapeutic effects for nasal mucosa regeneration in vitro and in vivo. In general, NTS was prepared by treating solutions with non-thermal plasma through a plasma device. NTS did not exhibit cytotoxicity toward BEAS-2B human bronchial epithelial cells while simultaneously enhancing cellular proliferation, migration and epithelial to mesenchymal transition during the wound healing process.

Furthermore, an in vitro study revealed that NTS also enhanced the matrix-metalloproteinase (MMP)-2/MMP-9 activities, which functioned to induce cell migration during wound healing. To provide more evidence on its efficacy, they tested NTS on rats by performing NTS-treated saline irrigation toward the nasal mucosa after the introduction of a wound at the right nasal septal mucosa. Epithelial thickness index (ETI) and sub-epithelial index (STI) were used to obtain the ratio of the average height of newly formed tissue in the treated group compared to the height of the control group. Histopathological analysis revealed that NTS significantly increased ETI, suggesting an increased epithelial proliferation while significantly decreasing STI, which translated into decreased edematous changes of the tissue. Furthermore, the NTS-treated group has less inflammatory cell infiltration compared to the control group. Taken together, this report has documented the potential efficacy of plasma therapy in enhancing the wound healing of nasal mucosa [88].

## 8. Conclusions

Over the past decades, many interventions in the post-trauma management of nasal mucosa healing have been innovated. With the advancement of medicine, a favorable outcome of sinus mucosa wound healing can be achieved. Besides the conservative treatment, stem cells and other regenerative medicine strategies offer great potential in airway epithelium regeneration. However, rigorous clinical trials need to be conducted to bring them to the mainstream treatment modality.

## Figures and Tables

**Table 1:** Effect of absorbable nasal pack on nasal wound healing parameters.

Absorbable Nasal Pack						
Study	Intervention	Control	Endoscopic	Adhesion	Granulation	Edema
[33]	Gelatin film	Unpacked	NA	=	=	NA
[34]	Gelatin foam	Unpacked	NA	=	=	=
[35]	Hyaluronan gel	Polyvinyl acetate (PVA)	NA	+	NA	NA
[36]	Hyaluronan gel	PVA	NA	=	NA	=
[38]	Hyaluronan gel	PVA	=	NA	NA	NA
[37]	Hyaluronan gel	Unpacked	NA	=	NA	=
[39]	Hyaluronan gel	Unpacked	+	+	NA	NA
[40]	Hyaluronan gel	Unpacked	NA	+	NA	NA
[41]	Fibrin glue	PVA	NA	NA	NA	+
[42]	Chitosan gel	Unpacked	=	NA	NA	+
[43]	Chitosan gel	Unpacked	=	NA	NA	+
[44]	Cellulose powder	PVA	+	+	NA	+
[45]	Potato starch	Gelatin-thrombin matrix	NA	NA	NA	=
[46]	Carboxymethyl cellulose	Potato starch	=	=	=	=
[47]	Polyurethane foam	Unpacked	+	NA	NA	+
[48]	Polyethylene glycol	Hyaluronan gel	NA	=	NA	=

NA (Not applicable); + (Favors intervention); = (Equivalent).

**Table 2:** Functional nasal pack for drug delivery.

Functional Nasal Pack			
Study	Nasal Pack	Drug Delivered	Outcomes
[49]	Silsos gel	Silver	Improves SNOT22 score and mucosal healing
[50]	SinuBandFP	Fluticasone propionate	Improves polyp score, adhesion, pain
[51]	Nasopore	Betamethasone	Improves healing and satisfaction
		Ciprofloxacin	Improves healing and satisfaction

## Abbreviations

ECM	Extracellular matrix
ESS	Endoscopic sinus surgery
SNOT22	Sino-Nasal Outcome Test 22
TCM	Traditional Chinese medicine
ZYD	Zhu-Yuan decoction
PMMA	Polymethyl-methacrylate
MSC	Mesenchymal stem cell
AEC	Alveolar epithelial cell
MSC-CM	MSC conditioned medium
SAEC	Small airway epithelial cell
HGF	Hepatocyte growth factor
NGF	Nerve growth factor
IGF-1	Insulin-like growth factor 1
NTS	Non-thermal plasma-treated solution
MMP	Matrix-metalloproteinase
ETI	Epithelial thickness index
STI	Sub-epithelial index
